# The Drink Question

**Published:** 1890-10-18

**Authors:** 


### Turning Over New Leaves.

### The Drink Question.

Every reader of The Hospital knows that a large proportion of the diseases and crimes which are most prevalent in England is due, directly or indirectly, to the use of intoxicating drinks. The records of our hospitals and dispensaries, and the statistics of our asylums, police courts, and prisons furnish sufficient evidence on this point so far as the poorer classes are concerned. While the experience of physicians and trained nurses among those who are wealthy or in easy circumstances is uniformly to the effect that the excessive use of alcohol is by no means confined to the poor, and that their services are often required to combat the results, not so much of gross intoxication as of the long continued daily drinking to slight excess of wines or liquors.

For the last forty years these facts have been brought before the public in many different ways—by the writings of physicians, the lectures and pamphlets of temperance reformers, the teachings of the pulpit, and most of all by the daily press. The magnitude of the evils due to the use of alcoholic drinks being thus admitted, and the cause of these evils being seemingly so well known and undisputed—why is it that an effectual remedy has not been found and applied?

The author of "The Drink Question"* presents no new facts and proposes no definite plan for lessening the evils; her work is merely a string of unauthenticated quotations to prove what very few people will deny, saying nothing that has not been said before with much more clearness, conciseness, and eloquence; and proposing nothing except that Government should interfere in some way. She does not advocate temperance properly so called, but total abstinence, at least, so far as individuals are concerned; but whether she would have Government compel everyone to be a total abstainer from malt liquors and wines, as well as from all forms of spirits, is by no means clear. This is not surprising, for as Mr. Pickwick remarked, in an unpublished address, it is very difficult for one person to convey to others ideas which he does not himself possess. Some idea of the style of the work may be obtained from the following sentence, which means almost precisely the reverse of what the author intended it should mean: "From whatever point of view we regard the temperance question, whether it be from the social, the domestic, the economic, or the scientific, the moral, mental, or physical, there we find it has struck deep roots to burrow away at and poison our natural life." (See page 238.)

Such books as this are not to be commended, for they are neither interesting, suggestive, or useful, yet everyone must sympathise with the motives which lead to their preparation and publication. Probably, every one who knows anything about the subject will agree that absolute prohibition of the sale of spirits, wines, and malt liquors in England is impossible. But is it possible to limit and restrict such sale—and, if so, how, and to what extent? Many schemes of legislation devised for this purpose have been tried in different parts of the United States, but none of them have met with such unqualified and undoubted success as to command general approval and assent. The best practical results seem to have been obtained by the system of high license combined with local option, but much depends on the character of the population, and especially as to whether it is largely urban in character. Prohibition in Maine has been to a certain extent a success, although liquor can be bought in any town in that State, and a gentleman can bring in wine and beer for his own use freely, but in Rhode Island and Massachusetts prohibitory legislation has been a total failure. We may be quite sure that any attempt to prevent by law the sale of beer in England or Germany, or the sale of wine in France, will not succeed until the character and customs of the great mass of the people have totally changed in many respects, for legislation on this subject must be strongly and continuously supported by public opinion if it is to produce any good results.

At the present time the great majority of the voters, legislators, and officials of this country are not in favour of, and do not practise, total abstinence, and have little respect for the opinions of those who urge it as a rule of life for all people. If progress is to be made in checking the excessive use of alcohol, it will probably be by legislation carefully considered with reference to the habits and wishes of the majority of the voting population in different localities—in other words, involving some form of local option—and it is in this direction that the efforts of the temperance reformers should be chiefly directed.

*The Drink Question: Its social and medical aspects. By Dr. Kate Mitchell. (London: Swan Sonnenschien and Co.)

### Domestic Hygiene.

"The object of the first part of this manual* is to place in the hands of the youth of both sexes, whether at home or in schools or colleges, a brief, fairly comprehensive, but above all practical account of the general construction and working of the human body and a summary and explanation of the laws of hygiene. The practical application of these laws in health and disease and the details of personal and domestic hygiene will form the subjects of Part II."

Such are the opening words of this excellent little treatise, and the author's aims have been well carried out. The style is clear and crisp, the reader going on easily and taking in with no difficulty the plain and straightforward account of the general facts of anatomy and physiology put before him. The author is very full of common sense, and one is constantly coming across a statement of which the contrary sense is often held by old-fashioned or ill-instructed people. We used to be told that everyone, including children, should leave off at a meal with a certain amount of appetite remaining. On this point our author says a healthy child should eat his fill at meal times. He, however, adds an important warning that no "in between meals" snacks should be allowed. Again, speaking of sugar, he emphasizes the value of it as a food stuff, but deprecates the constant eating of sweets at odd moments. The fact is, the old idea of sugar being bad for the teeth is exploded. The only form in which it does harm to the teeth is that in which it is taken too hard, with consequent danger of chipping the enamel, and so causing subsequent decay. It is astonishing how many people have been debarred from sugar from an idea of its harmfulness. For years it has been said to cause acidity, heartburn, and indigestion, and that it was particu-

* A Short Manual of Personal and Domestic Hygiene. By A. T. Schofield, M.D. (London: Allman and Son.)

larly prejudicial to gouty people, and it has taken all this time to arrive at the authoritative statement, which was made at a medical society's meeting this year (1890), that sugar might be eaten by gouty people. The fact of the matter is, that here and there one meets with a person to whom sugar is a poison, but the cases are exceptional. Of course those wishing to reduce their fat should not eat too much carbo-hydrate food in any form, nor should the unfortunate diabetic indulge in "best loaf." But the general public may dismiss the idea of the harmfulness of sugar, and eat it with a quiet mind.

Again, speaking of dress, the author gives full explanations of the reasons why this or that garment is better or worse than another. In one place he says two thin flannels are warmer than one thick one. Why? He gives the reason at once—a layer of warm non-conducting air between them. And so through the book we are constantly meeting with well-known facts which are very familiar to us, the explanation of which is not always obvious, but which our author explains and illustrates in the simplest and most direct manner. We strongly recommend this little work to all parents and teachers. Its tone is healthy, the knowledge it conveys is of the utmost importance, and the facts thus stated, if learned at the receptive period of youth, will bear good fruit as the learners take their part in the struggle of life.

Dr. Schofield also publishes a number of useful pamphlets (Religious Tract Society), called "Health at Home Tracts," at the price of one penny. These are more especially for the poorer classes, and are admirably adapted to teach them how to manage their small homes to the greatest advantage. The names will indicate their use: "How to be Healthy in One Room," "On the Care of Children," and so on. Finally, we note that, in these days of non-religious writing, Dr. Schofield is not afraid to state definitely his belief in the value of religion.

